# Congenital Perirectal Dermoid Cyst: A Rare Cause of Complex, Recurrent Pediatric Fistula-in-ano

**DOI:** 10.3389/fped.2018.00143

**Published:** 2018-05-16

**Authors:** Wesley E. Barry, Grace E. Asuelime, Shengmei Zhou, Jeffrey Hammoudeh, Henri R. Ford, Eugene S. Kim

**Affiliations:** ^1^Division of Pediatric Surgery, Children's Hospital Los Angeles, Los Angeles, CA, United States; ^2^Department of Surgery, Keck School of Medicine of the University of Southern California, Los Angeles, CA, United States; ^3^Department of Pathology and Laboratory Medicine, Children's Hospital Los Angeles, Los Angeles, CA, United States; ^4^Division of Plastic Surgery, Children's Hospital Los Angeles, Los Angeles, CA, United States

**Keywords:** dermoid cyst, fistula-in-ano, anal fistula, infection, pediatric surgery

## Abstract

Perianal abscess and fistula-in-ano are well-described in the pediatric population. They are most common in infants less than 1 year of age and often resolve with oral antibiotics; occasionally they require drainage or fistulotomy. The etiology is commonly associated with cryptoglandular obstruction and subsequent infection, however alternative diagnoses should be considered in cases of recurrent abscesses and fistulae that are refractory to standard treatments. In this report, we present the case of an 8-year-old boy with a complex, recurrent fistula-in-ano that resulted from a rare congenital perirectal dermoid cyst.

## Background

Perianal abscess and fistula-in-ano are relatively common in the pediatric population and are known to have a significant male predominance (1–8). Typically, these are believed to arise from abnormal crypts of Morgagni, which progress into a perianal abscess and in some cases, a fistula-in-ano (3, 9). This process most commonly occurs during infancy, specifically in males less than 1 year of age (2, 3). Conservative management of perianal abscess with antibiotic therapy appears to play a role in infants given the high rate of spontaneous resolution (1). However, in children older than 2 years of age, many surgeons recommend surgical drainage of perianal abscesses followed by post-operative antibiotics given the high risk of recurrent infections and development of fistula-in-ano following conservative management (1, 3–5, 7). It is important to consider that although this process most commonly is associated with cryptoglandular infection; inflammatory bowel disease, perianal/perirectal masses, and gastrointestinal (GI) duplications may present similarly (9–12).

## Case presentation

The patient is an 8-year-old boy with no significant past medical history who presented with a 2–3 year history of recurrent infections of the left buttock. These recurrent buttock abscesses were associated with localized pain, erythema, and foul-smelling discharge. The patient had received multiple courses of oral antibiotics with intermittent resolution of symptoms. There were no systemic signs of infection at the time of presentation. The patient was born full-term and had no significant health issues since birth and no previous surgeries. The patient's parents believe that he had a small left buttock dimple since infancy however only appreciated additional symptoms over the past 2 years.

On physical examination the patient is a well-appearing, active young boy. Heart, lung, and abdominal exam was unremarkable. There was a firm, 1 cm area of induration on the superomedial left buttock along the medial aspect of the left gluteal cleft. This area was tender to palpation however there was no significant erythema or fluctuance. Laboratory values at the time of presentation were within normal limits.

## Diagnosis and treatment

With a working diagnosis of a recurrent left gluteal abscess, the patient was taken to the operating room for surgical drainage of a left buttock abscess. In the operating room, the location of maximum fluctuance was incised with a scalpel, and a penrose drain was packed in the large abscess cavity and sutured to skin. Following this procedure, the patient continued to have a large amount of purulent drainage even after the drain was removed. Given the persistence of symptoms following incision and drainage, further work up was pursued.

**Figure 1:**
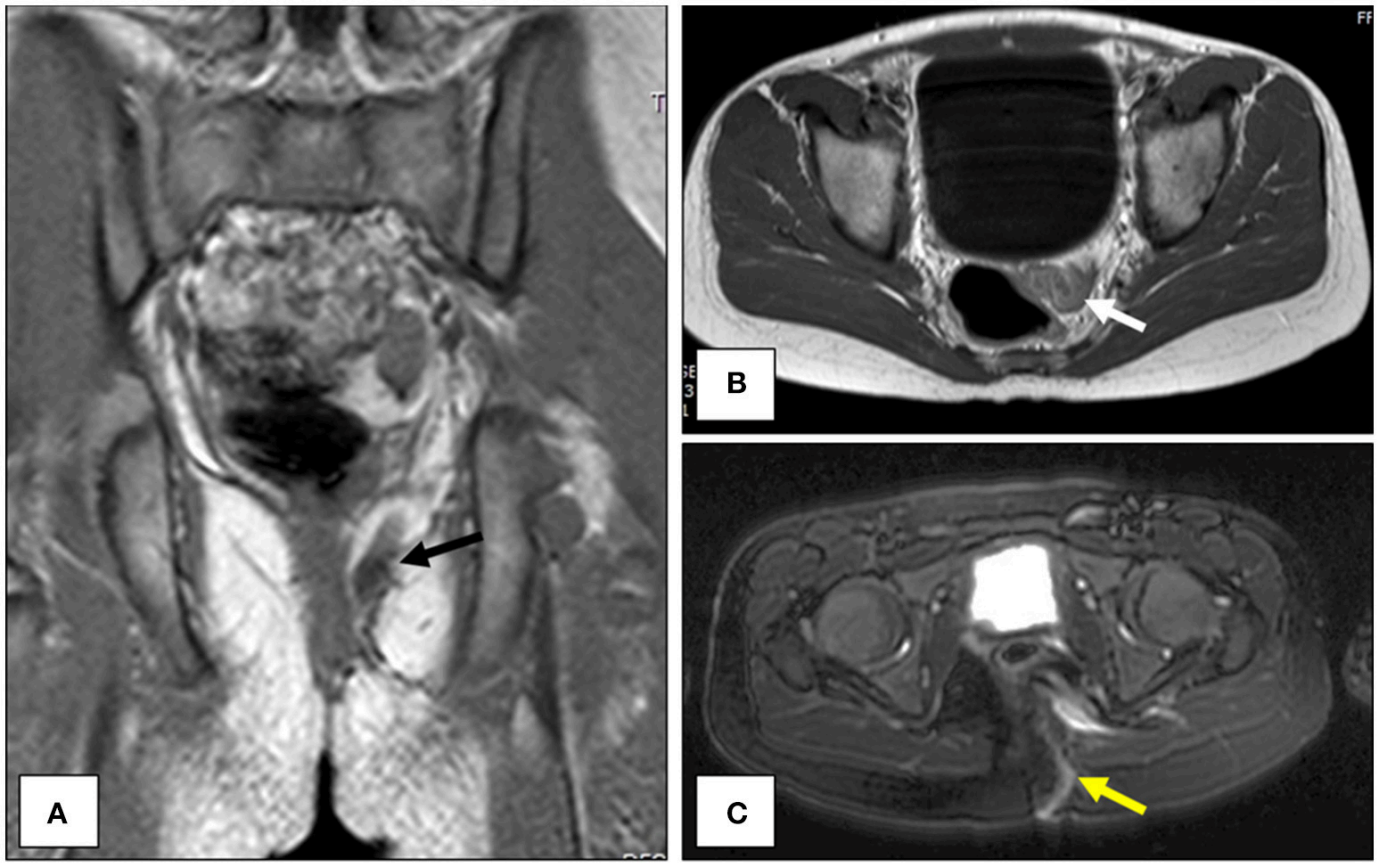
Magnetic resonance imagining (MRI) of the pelvis. The location of the suspected perirectal duplication is shown in the coronal (**A**, black arrow) and axial planes (**B**, white arrow). A T2-weighted image illustrating the course of the fistulous tract along the left gluteal musculature and out to the overlying skin (**C**, yellow arrow).

Magnetic resonance imagining (MRI) of the pelvis was obtained to evaluate for an underlying fistula, anomalous connection to a deeper abscess cavity or any indication of perirectal masses. The MRI revealed a small left superomedial gluteal abscess adjacent to the rectum with a tract extending through the left gluteus maximus muscle and extending superficially to the skin (Figure 1). The MRI was consistent with a superficial abscess with a fistulous tract toward the rectum, possibly due to a perirectal duplication. During this time, the patient was evaluated by the gastroenterology service and was found to have no risk factors for inflammatory bowel disease; laboratory evaluation revealed no evidence of systemic infection/inflammation (WBC count = 6,200 cell/μL, CRP <0.5 mg/dL, ESR = 7 mm/h) and markers of inflammatory bowel disease (serum IBD panel negative, fecal calprotectin <16 μg/g).

**Figure 2:**
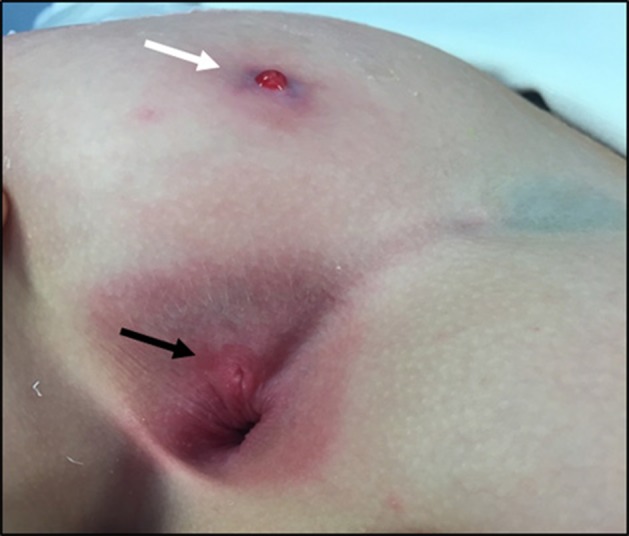
Photograph during examination at the time of the second operation. The area of original drainage and fistula opening along the superomedial aspect of the left buttock (white arrow) and the location of the perianal fistula opening (black arrow) are depicted.

**Figure 3:**
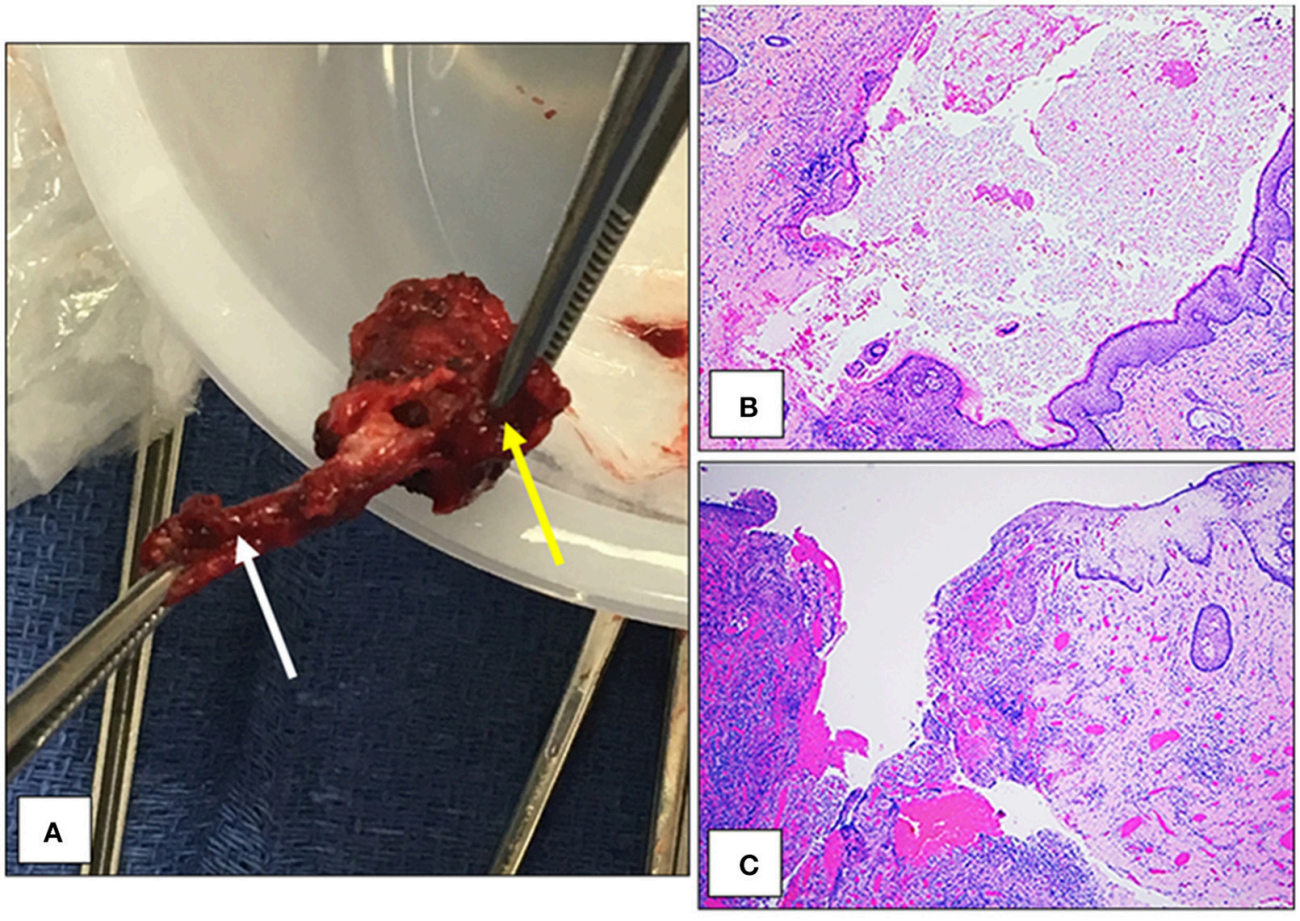
Gross and microscopic depictions of the congenital perirectal dermoid cyst. The cyst measured approximate 1 cm^3^ (yellow arrow) and had a well-developed fistulous tract (white arrow) **(A)**. Microscopically, there was a cyst lined by keratinizing stratified squamous epithelium with numerous hair follicles and sebaceous glands **(B)**. A fistula was identified that focally lined by keratinizing stratified squamous epithelium **(C)**.

The patient was taken to the operating room for a more thorough examination for a fistula via a parasagittal exposure. Prior to initiating the operation, our examination revealed a perianal opening in addition to the previous fistulous opening of the left buttock (Figure 2). To determine if these two fistulous openings were connected, we injected hydrogen peroxide into the original buttock opening and found bubbling fluid arise from the perianal opening, thus establishing a connection. Through a large parasagittal incision which traversed deep to the gluteus maximus muscle, we were able to identify an area of a large chronic abscess cavity in the deep gluteal region. We also discovered that both fistulous openings connected to this area, and we proceeded to debride and performed cautery fulguration of the perianal tract using a Bugbee cautery catheter. However following the operation, multiple infections of the wound and deeper spaces ensued, despite attempted gluteus rotational flap procedures. Lower barium enema studies and lower endoscopy studies failed to identify a connection from the rectum to the wound. Given persistent infections without clear cause, it was discussed with the patient's parents to proceed with a more extensive dissection of the perianal and perirectal space to evaluate for the source of these recurrent infections as well as a diverting colostomy for fecal diversion. He therefore was taken to the operating room, and the dissection began with suture control of the anal fistula opening. Proximal dissection of the tract was performed with suture retraction which led to a cystic structure with hair and solid components approximately 3–4 cm proximally. The entire tract and cyst were excised en bloc and passed off the field for pathology examination (Figure 3A). The cavity was irrigated, a drain was placed and a diverting colostomy was created given the extent of the dissection adjacent to the rectum. The patient tolerated the procedure well and without complication.

Pathologic evaluation of the mass grossly revealed a 3.5 × 1.3 × 1.0 cm mass with a cystic structure measuring 1.7 × 0.5 cm, filled with sebaceous material and hair (Figure 3A). Microscopically, the cyst contained keratin and hair shafts (Figure 3B). In addition the cyst was focally ruptured and replaced by granulation tissue formation, acute and chronic inflammation, and focal foreign body giant cell reaction (Figure 3C). There was no evidence of malignancy.

Following the removal of the congenital perirectal dermoid cyst, the patient has had no infectious recurrences. He has since had his colostomy taken down without complication and is back participating in school and everyday activities.

## Discussion

The presentation of a congenital dermoid cyst in the perirectal location in a child is rare, and there is no previously reported pediatric case report in the literature. Two similar cases of adults presenting with complex fistula-in-ano secondary to dermoid cysts have been reported, however these cysts were discretely located in the presacral/retrorectal space (10, 11). This retrorectal space is bordered superiorly by the peritoneal reflection, inferiorly by the supralevator muscle complex, anteriorly by the rectum, posteriorly by Waldeyer's presacral fascia and laterally by the iliac vessels (10). Multiple studies have shown this to be a rare but important location for the development of numerous tumor types including developmental cysts such as dermoid cysts (10, 11). Despite these previous reports, the dermoid cyst in our case appeared to be in the intersphincteric space as it was more caudal than the location described in these case reports. Additionally, a perirectal mass in this location may represent a rectal duplication. GI duplications are defined by the Ladd's criteria in which a mass must be (1) near or in proximity to the GI tract, (2) have a layer of smooth muscle, and (3) covered by GI mucosa (13, 14). The mass in our case was in proximity to the rectum but did not have either of these histologic findings consistent with a rectal duplication.

Here we present the case of a congenital perirectal dermoid cyst that presented as a complex, recurrent fistula-in-ano in a child. Perianal abscess and fistula-in-ano are a relatively common disease process encountered by pediatric surgeons (1, 4). These are most common in infant males less than 1 year of age and are thought to be caused by abnormal development and subsequent infection of the crypts of Morgagni (4, 9). However, in rare cases of recurrent and refractory infections, one should consider alternative diagnoses including, but not limited to developmental cysts such as a congenital dermoid cyst.

## Ethics statement

Written informed consent for the publication of this case report was obtained from the parents of the patient.

## Author contributions

WB this author has been involved with the care of this patient. The author played a primary role in drafting and revising the manuscript. GA this author assisted in the review of this case and has played a significant role in the drafting and revision of the manuscript. SZ this author assisted with the pathologic review of this case and has played a significant role in the drafting and revision of the manuscript. JH this author played a key role in the management of this patient. The author has assisted in the drafting and revision of the manuscript. HF this author played a key role in the management of this patient. The author has assisted in the drafting and revision of the manuscript. EK this author played a primary role in the care of this patient. The author has supervised the drafting and revision of this manuscript. All authors have reviewed and given final approval of this version to be published.

### Conflict of interest statement

The authors declare that the research was conducted in the absence of any commercial or financial relationships that could be construed as a potential conflict of interest.
